# List of Diseases from the 20th of July to the 20th of August; Being the Result of the Public and Private Practice of a Physician at the West End of the Town

**Published:** 1799-09

**Authors:** 


*List of Diseases from the 20th of July to the 20th of August; being the Result of the Public and Private Practice of a Physician at the West End of the Town.*

| | | | |
|---|---|---|---|
| ACUTE DISEASES. | | Pyrosis | 2 |
| Measles | 9 | Enterodynia | 5 |
| Scarlatina | 1 | Diarrhœa | 13 |
| Small-pox | 1 | Cholera and Bilious Vomiting | 3 |
| Hooping-cough | 3 | Colica Pictonum | 1 |
| Contagious malignant Fever | 7 | Hæmorrhoids | 4 |
| Synochus, or Summer-fever | 4 | Fluor albus | 5 |
| Slow fever and Hectic | 4 | Menorrhagia | 5 |
| Catarrh | 6 | Amenorrhea, &c. | 7 |
| Acute Rheumatism | 2 | Jaundice | 2 |
| Erysipelas | 1 | Scirrhus of the Liver | 1 |
| Inflammatory Sore-throat | 3 | Scirrhus of the Womb | 2 |
| Child-bed and Milk-fevers | 3 | Prolapsus | 1 |
| Acute Diseases of Infants | 8 | Dysury | 1 |
| CHRONIC DISEASES. | | Worms | 2 |
| Asthenia | 28 | Rickets | 3 |
| Chronic Rheumatism | 10 | Scrophula | 4 |
| Dropsy | 8 | Tabes Mesenterica | 2 |
| Paralysis | 2 | Lichen | 2 |
| Apoplexy | 1 | Lepra | 1 |
| Cephalæ | 5 | Scaly Tetter | 3 |
| Hysteria | 2 | Urticaria | 1 |
| Epilepsy | 1 | Roseola | 1 |
| Cough and Dyspnœa | 15 | Erythema | 1 |
| Hæmoptoe | 2 | Ringworm | 1 |
| Pleurodyne | 1 | Itch and Prurigo | 13 |
| Pulmonary Consumption | 5 | Porrigo | 2 |
| Dyspepsia | 12 | Acne | 3 |
| Gastrodynia | 7 | Lupus | 1 |

The long continuance of wet and cold weather has not produced in the course of the two last months, any considerable extension of diseases. The measles have been the prevailing contagious epedimic; and, contrary to the position of Sydenham, have spread more widely since the solstice. The disease has however appeared in a very mild form; as it usually does in the summer months, with but a slight cough, and little fever.

The malignant contagious fevers occured in the close and crowded dwellings of the poor. That the number of them should be greater than usual, was probably owing to the long series of damp and windy days, which confined most of the individuals of families at home, and prevented ventilation, by open windows, &c. Nevertheless induction, from the experience of many years, enables me to ascertain, that mild open winters, and chill, even though moist summers, are on the whole, most favourable to the health of the inhabitants of London.

